# Supplementary material for: Association between levels of trimethylamine N-oxide and cognitive dysfunction: a systematic review and meta-analysis
Source: PeerJ. 2025 Sep 5;13:e20000. doi: 10.7717/peerj.20000 (PMC12422268; doi:10.7717/peerj.20000)
Supplement: Supplemental Information 2 [file peerj-13-20000-s002.docx]

The audience of this meta-analysis is the following groups：

Clinical Researchers:

• Neuropsychiatrists investigating dementia prodromal markers

• Endocrinologists studying metabolic-cognitive correlations

Basic Scientists:

• Microbiome-metabolome interaction specialists

• Lipidomics platform developers
